# Does stimulus predict the best candidates for deep brain stimulation for PD?

**DOI:** 10.1186/1745-6215-14-S1-P114

**Published:** 2013-11-29

**Authors:** Caroline Rick, Smitaa Patel, Natalie Ives, Francis Dowling, Jane Daniels, Steven Gill, Crispin Jenkinson, Rosalind Mitchell, Hardev Pall, Niall Quinn, Carl Clarke, TRK Varma, Keith Wheatley, Adrian Williams

**Affiliations:** 1 University of Birmingham, Birmingham, UK; 2 Frenchay Hospital, Bristol, UK; 3 University of Oxford, Oxford, UK; 4 Queen Elizabeth Hospital, Birmingham, UK; 5 National Hospital for Neurology and Neurosciences, London, UK; 6 Sandwell and West Birmingham Hospitals NHS Trust, Birmingham, UK; 7 The Walton Centre, Liverpool, UK

## Background

Deep Brain Stimulation (DBS) is used for people with Parkinson's disease (PwPD) who are experiencing motor complications that are not controlled by medication. To date, PD SURG is the largest trial to compare DBS to best medical therapy in PwPD.

Outcomes to DBS for PwPD are varied and there is an ongoing question as whether patient selection can be improved. To date, subgroup analyses have not been powered to provide an answer. An algorithm (STIMULUS) was designed to try to identify suitable candidates for DBS for PD symptoms based on the clinical opinion of an expert panel.

The PD SURG data set provides an opportunity to test the ability of STIMULUS to predict suitable candidates.

## Aims

To test the STIMULUS algorithm's ability to predict surgical outcomes for PwPD.

## Methods

Pre-surgical characteristics from the PD SURG trial together with data from clinician-rated UPDRS data were mapped on to the STIMULUS clinical variables from which the STIMULUS programme algorithm calculated which patients would be deemed "suitable", "uncertain" or "unsuitable" for referral for DBS. These predictions were compared to the change in patient-rated quality of life (QoL) and UPDRS between the baseline and 1 year data.

## Results

Results from 277 PD SURG participants did not show any correlation between STIMULUS predictions and changes in QoL or UPDRS 1 year post-surgery.

## Conclusions

STIMULUS does not predict the most suitable candidates for DBS. Its use may, therefore, reject suitable candidates for DBS.

